# The Effect of Larger Orthodontic Forces and Movement Types over a Dental Pulp and Neuro-Vascular Bundle of Lower Premolars in Intact Periodontium—A Numerical Analysis

**DOI:** 10.3390/dj12100328

**Published:** 2024-10-14

**Authors:** Radu-Andrei Moga, Cristian Doru Olteanu, Ada Gabriela Delean

**Affiliations:** 1 Department of Cariology, Endodontics and Oral Pathology, School of Dental Medicine, University of Medicine and Pharmacy Iuliu Hatieganu, Str. Motilor 33, 400001 Cluj-Napoca, Romania; ada.delean@umfcluj.ro; 2 Department of Orthodontics, School of Dental Medicine, University of Medicine and Pharmacy Iuliu Hatieganu, Str. Avram Iancu 31, 400083 Cluj-Napoca, Romania

**Keywords:** dental pulp, neuro-vascular bundle, ischemic risks, intact periodontium, orthodontic force, finite element analysis, orthodontic movement

## Abstract

Background/Objectives: This numerical analysis of stress distribution in the dental pulp and neuro-vascular bundle (NVB) of lower premolars assessed the ischemic and degenerative–resorptive risks generated by 2 and 4 N during orthodontic movements (rotation, translation, tipping, intrusion and extrusion) in intact periodontium. Methods: The numerical analysis was performed on nine intact periodontium 3D models of the second lower premolar of nine patients totaling 90 simulations. Results: In intact periodontium, both forces displayed a similar stress distribution for all five orthodontic movements but different amounts of stress (a doubling for 4 N when compared with 2 N), with the highest values displayed in NVB. In intact periodontium, 2 N and 4 N induced stresses lower than the maximum hydrostatic pressure (MHP) with no ischemic risks for healthy intact teeth. The rotation was seen as the most stressful movement, closely followed by intrusion and extrusion. Translation was quantitatively seen as the least stressful when compared with other movements. Conclusions: Larger orthodontic forces of 2 N and 4 N are safe (with any expected ischemic or resorptive risks) for the dental pulp and NVB of healthy intact teeth and in intact periodontium. Nevertheless, rotation and translation movements can induce localized circulatory disturbances in coronal pulp (i.e., vestibular and proximal sides) generating ischemic and resorptive risks on previously treated teeth (i.e., direct and indirect dental pulp capping). The intrusion and extrusion movements, due to the higher NVB-induced deformation when compared with the other three movements, could trigger circulatory disturbances followed by ischemia on previously traumatized teeth (i.e., occlusal trauma).

## 1. Introduction

Due to local anatomy, dental pulp and neuro-vascular bundle (NVB) are prone to suffer from circulatory disturbances and ischemia leading to necrosis and pulpitis during orthodontic treatment. Moreover, if the tooth was previously traumatized and/or injured (i.e., occlusal trauma) [1,2] or suffered from various dental treatments involving dental pulp (i.e., direct and indirect pulp capping) [3,4,5,6], its reactivity and tissular adaptability will diminish.

Much of the stresses induced by the orthodontic loads during the movements are absorbed and dissipated by the periodontal ligament (PDL) surrounding the tooth and protecting the pulp and NVB [3,4,5]. Anatomically speaking, NVBs are found in the apical third of the PDL (directly subjected to the orthodontic stresses), while dental pulp is found inside the tooth (i.e., in the pulp chamber and root canals) [3,4,5]. The PDL is rich in circulatory vessels, enabling the blood supply and metabolism for the PDL, dental pulp and bone [3,4,5]. The physiological circulatory pressure (ensuring the proper metabolism) at this level is 16–22 KPa (maximum hydrostatic pressure (MHP), about 80% of the systolic pressure) [3,7,8,9,10].

Biomechanically, in a healthy periodontium, the orthodontic movement is triggered by small and limited circulatory disturbances induced by orthodontic loads (i.e., in the PDL, NVB and dental pulp), resulting in PDL and bone remodeling [4,11,12]. Nevertheless, if the applied orthodontic loads produce quantitative stresses exceeding the MHP (local circulatory vessels collapse under increased pressure) for longer periods, these induced circulatory disturbances will exponentially increase the ischemic and resorptive risks [3,13,14]. Thus, both the type and amount of load, as well as appliance time, have a direct influence over the circulatory disturbances and degenerative processes happening in the dental pulp and NVB [3,4,5,15,16,17]. Moreover, if the tooth and periodontium were previously injured and/or traumatized [15,17,18], pulpal and NVB structural and functional alterations are induced [6,19,20,21,22,23,24,25,26,27,28], with increased ischemic and degenerative–resorptive risks when compared with healthy periodontium [3,4,5].

It must be emphasized that dental pulp and NVB cannot be directly inspected and studied; thus, any morphological and functional alterations could be seen only lately when they already produced the clinical symptomatology of pulpitis and its complications [15,16,17,18,28,29,30,31,32,33,34,35]. However, indirectly, through numerical studies displaying the stresses induced by orthodontic loads during the movements, the ischemic and resorptive risks could be evaluated [3,4,5].

The use of light orthodontic forces of around 0.5–1 N was reported to be safe in intact periodontium by both clinical and numerical studies [4,36,37,38,39] but with reports of ischemia and regressive changes when a larger amount is used [11,12,40,41]. However, the subject of optimal orthodontic force (light or large) is still a subject of debate, with it being accepted that there is a need for more data [3,4,5]. Even more confusing are the reports about the poor quality of multiple in vivo studies (due to methodological errors) supporting the need for new studies (with correct methodology and clinical correlations) focusing on stress distribution [12,30,40]. There are few numerical studies investigating the dental pulp and NVB available in the current research flow (some of them belonging to our team [4,5,37,38,39]), but there are more assessing the PDL (especially the apical third holding the NVB). However, most PDL studies provide confusing and contradicting reports due to their methodological errors (as explained in our previous studies, the first to address this issue and providing a correct method for accurate results [4,5,37,38,39,42,43,44]). Thus, recent numerical PDL studies reporting 0.28–4 N to be safe for orthodontic movements in intact periodontium [7,8,45] totally contradict the reports backing the light forces [4,5,36,37,38,39]. Other recent numerical studies (by correctly employing the finite element method) brought new data about the high ability of periodontal tissues to sustain damages without major functional alterations [42,43], backing up the idea of applying more than 1 N and reporting less amounts of stress displayed at the pulp–NVB level.

Despite the many advantages of numerical studies for reporting accurate results (as in the engineering field), some mandatory issues must be addressed (i.e., failure criteria, boundary assumptions, sample size and anatomical accuracy) as proven in previous studies (the first to address these issues) [3,4,5,37,38,39,42,43,44,46]. None of the previously available numerical studies addressed the accuracy-related mandatory issues, providing results with debatable accuracy (i.e., reports that sometimes contradicted clinical data and exceeded physiological MHP even for light forces) [7,8,9,10,15,16,17,18,30,46,47,48,49,50,51,52,53,54,55,56,57,58,59,60,61,62]. Nonetheless, without properly addressing the boundary assumption issue, the current available numerical studies [7,8,9,10,15,16,17,18,30,46,47,48,49,50,51,52,53,54,55,56,57,58,59,60,61,62] employed the correct linear elasticity, isotropy and homogeneity for dental tissues, as proven by other reports [3,4,5,37,38,39,42,43,44,46].

Thus, to obtain detailed information about the stress distribution in dental pulp and NVB (too small and complex structures impossible to be in vivo examined), the only available and accurate method is the numerical analysis, performed over 3D models that were CBCT (cone-beam computed analysis) based [3,4,5,37,38,39,42,43].

This study is part of larger stepwise research (with clinical protocol 158/02.04.2018) that is continuing the study of orthodontic force effects over dental tissues in various degrees of periodontal loss [4,5,37,38,39,42,43,44]. Our previous studies are the first to report the accuracy issues related to the employment of the finite element method in dentistry, as well as the correct protocol for fixing them.

To have new data about the biomechanical behavior of larger orthodontic forces over the dental pulp and NVB, the aims of this study were to numerically assess the stress distribution of 2 N and 4 N in the two above-mentioned structures during the five most-used orthodontic movements (i.e., rotation, translation, tipping, intrusion and extrusion) and in intact periodontium. Additionally, the assessment of ischemic and degenerative–resorptive risks was performed by correlations with the physiological MHP.

## 2. Materials and Methods

This research focuses on dental pulp and neuro-vascular bundles in intact periodontium and was conducted over nine 3D models of the second lower premolar, totaling 90 numerical simulations. The selected sample size was nine (nine patients, four males/five females, mean age 29.81 ± 1.45) as in our earlier studies [4,5,37,38,39,42,43,44].

The inclusion criteria: no missing teeth in the investigated region, no malposition involving the analyzed tooth, intact premolar free of any dental treatments (endodontic/filling/crown), bone loss of maximum 1–2 mm, healthy periodontium, orthodontic treatment indication and proper oral hygiene.

The exclusion criteria: particular root geometry (e.g., non-fused double root, angulated root, extreme curvature, etc.), abnormal crown shape with multiple cusps, temporary teeth, abnormal root surface defects (e.g., external root resorption), bone defects (radiologically visible bone defects), abnormal pulp chamber and root canals (e.g., internal resorption), more than 2 mm bone loss and poor oral hygiene after acceptance with visible signs of inflammation.

The region of interest has been radiologically investigated using a CBCT (ProMax 3DS, Planmeca, Helsinki, Finland; voxel size of 0.075 mm) and contained the 2nd lower premolar with two adjacent teeth.

**Figure 1:**
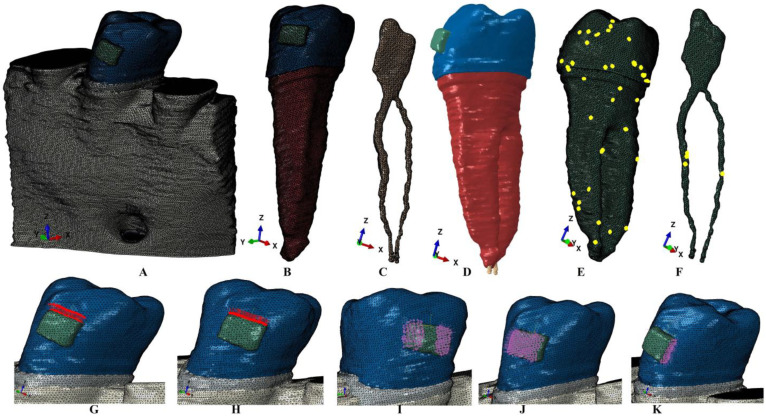
Mesh model (of one of the nine 3D models): (**A**)—2nd lower right premolar model with intact periodontium, (**B**)—second lower premolar, (**C**)—dental pulp and NVB, (**D**)—premolar with dental pulp and NVB, (**E**)—second lower premolar with mesh elements warnings, (**F**)—dental pulp and NVB mesh with element warnings; applied vectors: (**G**)—extrusion, (**H**)—intrusion, (**I**)—rotation, (**J**)—tipping, (**K**)—translation.

The manual software reconstruction (due to anatomical complexity of the region) process employed Amira 5.4.0 (Visage Imaging Inc., Andover, MA, USA). Each tissular part (i.e., enamel, dentine, dental pulp, neuro-vascular bundle (NVB), periodontal ligament (PDL) and trabecular and cortical bone) was individually identified and selected (Figure 1). Because of the similarity of physical properties (Table 1) and DICOM appearance, the cementum was reconstructed as dentine. The PDL’s natural variable thickness of 0.15–0.225 mm was guarded and held the NVB.

In each of the nine 3D models with only limited bone loss, the second lower premolar was guarded, while the rest of the alveolar bone was filled with trabecular and cortical bone. The missing bone and PDL were reconstructed obtaining nine intact periodontium 3D models used in our study. A stainless-steel bracket base was reconstructed on the vestibular side of the crown to avoid any potential interference due to various bracket designs.

The mesh had 5.06–6.05 million C3D4 tetrahedral elements and 0.97–1.07 million nodes, while the global element size was 0.08–0.116 mm (Figure 1). Due to manual reconstruction, the mesh models displayed few element warnings (e.g., tooth displayed 39 element warnings—0.00589% for a total of 661,137 elements (Figure 1E), while pulp and NVB showed 4 element warnings—0.0158% for a total of 25,252 elements (Figure 1F)—but no element errors. It must be emphasized that element warnings are placed in non-essential regions, with quasi-continuity in essential areas, and all internal checking algorithms were successfully passed.

The numerical analysis employed Abaqus 6.13–1 (Dassault Systèmes Simulia Corp., Maastricht, The Netherlands) software. Five of the most common orthodontic movements (rotation, translation, tipping, intrusion and extrusion) under 2 N and 4 N applied force were investigated. The Tresca failure criterion (i.e., maximum shear stress) proper for analyzing dental tissues of ductile resemblance was applied [4,5,37,38,39,42,43,44]. The boundary assumptions were related to encastered base of the models, perfectly bonded interfaces, linear elasticity, isotropy and homogeneity as in previous numerical studies [4,5,37,38,39,42,43,44].

The stress distribution was displayed as color-coded projections (red–orange high, yellow–green moderate, blue low) and was correlated with the 16–22 KPa of the physiological maximum hydrostatic pressure to be able to evaluate the ischemic risks induced by larger orthodontic forces in intact periodontium.

## 3. Results

**Figure 2:**
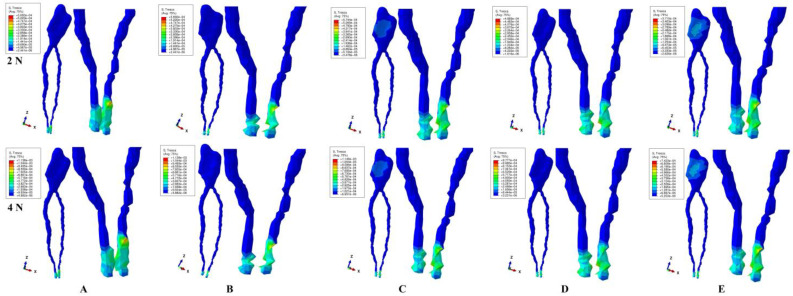
Comparative stress distribution for 2 N and 4 N applied force (of one of the nine 3D models) in intact periodontium: (**A**)—extrusion, (**B**)—intrusion, (**C**)—rotation, (**D**)—tipping, (**E**)—translation.

During all five movements and two forces applied in intact periodontium, the highest amounts of stress were seen at the NVB level while the rest of the dental pulp was subjected only to limited amounts of stress (Figure 2 and Table 2). Thus, during orthodontic movements, the NVB was prone to higher ischemic risks than the dental pulp (which anatomically is protected by the dental pulp chamber and root canals). Qualitatively, the two applied orthodontic forces displayed similar stress distribution, with only quantitative differences (a doubling of numerical values for 4 N when compared with 2 N).

Nevertheless, all displayed amounts of stress (i.e., Table 2—the average amount of stress for all nine patients) were lower than the MHP (i.e., of 16 KPa), making it seem that 4 N of orthodontic force does not induce any ischemic and resorptive risks on the NVB and dental pulp of the healthy intact premolars and in intact periodontium. Thus, the stress amount in the NVB induced by 4 N of applied rotation is 21.6 times lower than the MHP.

Among the five orthodontic movements, rotation induced the highest amounts of stress, closely followed by intrusion and extrusion (Table 2), while translation and tipping were the lowest. Thus, rotation seems to be the most stressful among the five orthodontic movements.

Qualitatively (i.e., the color-coded stress display, Figure 2), most of the NVB stress areas for all five movements are color-coded in yellow–green (moderate intensity) with only limited areas of orange–red (high intensity) being prone to localized circulatory disturbances. Quantitatively, the highest stresses (i.e., the average for the nine patients, lower than the 16 KPa) are displayed in Table 2, with no ischemic risks prone to induce pulp necrosis or tissular resorption in healthy intact teeth.

The rotation and translation movements seem to induce coronal pulp stress (various shades of color-coded blue areas, low intensity (Figure 2C,E), on vestibular, mesial and distal sides of the coronal pulp) lower than the MHP. However, these are prone to localized circulatory disturbances, with impact on previously treated teeth (direct/indirect pulp capping on the proximal sides of the dental pulp). Despite being lower than the physiological 16 KPa, the localized circulatory disturbances might induce pulpal risks (i.e., leading to pulpitis) especially induced by translation movements. The other three movements seem not to affect the coronal pulp.

The root canal pulps seem not to be influenced by the amount of applied orthodontic force or by the type of orthodontic movement.

The NVB area seems to bear the highest physical deformation during the intrusion and extrusion movements (Figure 2A,B) when compared with the other three movements, with potential degenerative and ischemic risks on previously traumatized teeth.

## 4. Discussion

This numerical analysis performed on second lower premolars and totaling ninety simulations revealed that, in intact periodontium, larger orthodontic forces up to 4 N of the applied load showed little influence over the dental pulp and NVB of healthy intact teeth. Thus, quantitatively, the maxim stress amount displayed in the NVB by the rotation (the most stressful among the five movements and closely followed by intrusion and extrusion) was up to twenty-one times lower than the MHP, while the effect over dental pulp is even less important.

These results are similar with our earlier reports about the same issues but employ light orthodontic forces (using the same boundary assumption, movements, and 3D models of the second lower premolar) [4,5], as well as with other in vivo [15,16,17,18,30] reports and Proffit et al. [36]. Moreover, our findings are supported by our previous reports regarding the absorption–dissipation ability of tooth and periodontal ligament, reporting that only an extremely small percentage of the applied loads reached the pulp and NVB [3,4,5,37,38,39,42,43]. Thus, since the apical third of PDL holds the NVB and one of the major roles is of load absorption–dissipation, our results are biomechanically correct and in line with clinical data [3,4,5,37,38,39,42,43]. Nevertheless, since our quantitative results (Table 2) are lower than 16 KPa, no ischemia and degenerative–resorptive risks can be predicted in healthy periodontium and intact teeth (in line with our above-mentioned previous studies and common clinical knowledge). However, since the PDL receives the most stress amounts, further numerical simulations are needed to assess the periodontal ligament stress distributions under large loads.

However, if the tooth was previously injured/traumatized (i.e., occlusal trauma) [6,15,17,18,19,20,21,22,23,24,25,26,27,28] and/or the dental pulp had suffered injuries during dental treatment (i.e., direct or indirect pulp capping) [15,16,17,18,28,29,30,31,32,33,34,35], both NVB and dental pulp could suffer from ischemia and degenerative–resorptive risks in line with other studies [1,2]. Thus, rotation and translation are prone to produce circulatory disturbances leading to ischemia and degenerative–resorptive risks at the coronal pulp level (stress display on vestibular, mesial and distal sides), as seen in Figure 2C,E, in line with other studies [6,19,20,21,22,23,24,25,26,27,28]. The intrusion and extrusion movements due to a higher stress deformation of the NVB (Figure 2A,B) are prone to circulatory disturbances, ischemia and degenerative and resorptive risks, as reported by Minch et al. [14] and other studies [15,16,17,18,28,29,30,31,32,33,34,35].

Related to the above, it must be remembered that dental pulp and NVB functional alterations also appear during aging due to various degenerative–resorptive processes that cannot be evaluated and quantified by in vivo means, increasing the ischemic risks when employing large forces [1,15,18,29]. The current dental treatment that involves direct and indirect pulp capping induces local functional and morphological alterations [1,6], with impact on long-term tissular reactivity under functional loads [15,16,17,18,28,29,30,31,32,33,34,35].

The rotation was displayed as the most stressful movement among the five assessed, as in our earlier reports [3,4,5,37,38,39,42,43], and closely followed by intrusion and extrusion (Table 2), in line with Minch et al. [14] and Hofman et al. [9,10] who reported intrusion to be the most stressful in intact periodontium.

There are few data available about the biomechanical behavior of dental pulp and NVB during orthodontic movements due to the difficulties in reconstructions of such small and complex structures. Nevertheless, since the PDL apical third anatomically holds the NVB, correlations with its functional biomechanical reports could be performed. However, most numerical studies reconstructed the apical third PDL without its proper NVB [7,8,9,10,45]. Thus, various amounts of stress have been reported that usually exceeded MHP even in the case of the light orthodontic forces (e.g., Hofman et al. [9,10] reported for 1 N of intrusion 80 KPa, and 3–6 N lingual torque around 40 KPa, signaling not only circulatory disturbances but also extensive degenerative–resorptive processes that clinically are not happening). Wu et al.’s numerical studies [7,8,45] assessed the optimal orthodontic force, reporting 2.1–2.9 N of optimal rotation for a premolar (with stresses lower than MHP at PDL apical third level, and no stress at cervical level—biomechanically incorrect), contradicting clinical and numerical data [15,16,17,18,30,48,49], as well as Proffit et al. [36], who reported that the safest to be used are the light forces. Our reports, being based on better anatomical accuracy, provide better results when compared with other numerical analyses [9,10,15,16,17,18,30,47,48,49,50,51,52,53,54,55]. Nevertheless (a limitation of the method), it must be emphasized that our analysis was performed with pure orthodontic movements, but, in clinical reality, there is often associations of movements that in fact could reduce the applied loads, thus the numerical results, as well as the qualitative stress distribution, could be in fact clinically a little bit lower. However, numerical studies are the only available method to see the stress distribution in such complex structures, since in vivo offers only a general display of the entire structure.

About the numerical studies, despite their proper suitability for small and complex tissues (as herein), some issues that directly influence their accuracy must be discussed [9,10,47,48,49,50,51,52,53,54,55]. Thus, the selection of the proper failure criteria, boundary assumptions and anatomical accuracy are mandatory, as well as the sample size, which, if not applied, are seen as serious limitations of the method.

The failure criteria are selected based on the type of materials, reported as mandatory for accurate results [46]. Previous studies [3,4,5,37,38,39,42,43] of our team reported the ductile resemblance of the living dental tissues and Tresca being the suitable criteria for accurate numerical studies. Most of the previous numerical studies [9,10,15,16,17,18,30,47,48,49,50,51,52,53,54,55] ignored this mandatory aspect, providing reports with accuracy issues signaled by our studies [3,4,5,37,38,39,42,43].

The boundary assumptions are related especially to the isotropy, linear elasticity and homogeneity that were often used in numerical studies, despite the living tissues behaving in the opposite way. Nonetheless, these assumptions were proven to be correct up to 2.4 N by our previous reports [3,4,5,37,38,39,42,43], but only if Tresca criteria was employed (i.e., was engineeringly designed for non-homogeneity and anisotropy, while the use of small loads ensured small tissular displacements for meeting linear elasticity).

The anatomical accuracy of the analyzed 3D models was directly influenced by the number of elements and nodes (6.05 million tetrahedral elements, 1.07 million nodes, global element size of 0.08–0.116 mm, 40–12,731 times more elements, 4.4–1463 times more nodes than previous studies [9,10,15,16,17,18,30,47,48,49,50,51,52,53,54,55]) as well as the type (based on CBCT images and not on idealized simplified models as most other previous numerical analyses) and the number of modes (sample size, nine herein vs. one in previous).

The premolar mandibular region was selected since most of the current numerical studies assess the molar and incisor regions, and the data about the premolar region are scarce despite being placed in an occlusal support region and taking part in all functional processes.

The sample size for the numerical study method is usually one (specific to engineering); thus, most of the dental studies used one patient/one model and few simulations [46,48,49,52,54,56,57,58,59,60,61,62] vs. here being nine. It must be emphasized that this method developed in the engineering field requires only one model due to specific possibilities to cover varying experimental conditions leading to different results [5,7,8,38,39,46,56,57,58,59,62].

Nevertheless, to enhance the result accuracy, our research employed a sample size of nine with a total of ninety simulations. Thus, by following all the above-mentioned mandatory conditions, our numerical analysis ensured accurate results for dental tissues, as in the engineering field. However (a limitation of the method), numerical analyses cannot accurately meet all the clinical conditions; thus, they need to be validated by correlation with clinical data (e.g., MHP), while the results need to meet the clinical knowledge. Future research needs to be focused on the effect of larger orthodontic forces during the periodontal breakdown process and assess the same risks as herein.

## 5. Conclusions

1. In intact periodontium, both forces displayed similar stress distribution for all five orthodontic movements but different amounts of stress (a doubling for 4 N when compared with 2 N), with the highest values displayed in the NVB.
2. In intact periodontium, 2 N and 4 N induced stresses lower than the MHP with no ischemic or degenerative–resorptive risks for healthy intact teeth.
3. Rotation was seen as the most stressful movement, closely followed by intrusion and extrusion, while translation was the least stressful.
4. The rotation and translation movements can induce localized circulatory disturbances in coronal pulp (i.e., vestibular and proximal sides), generating ischemic and degenerative–resorptive risks on previously treated teeth (i.e., direct and indirect dental pulp capping).
5. The intrusion and extrusion movements, due to the higher NVB-induced deformation when compared with the other three movements, could trigger circulatory disturbances followed by ischemia and degenerative–resorptive risks on previously traumatized teeth (i.e., occlusal trauma).

## 6. Practical Implications

There are only a few studies available that investigate the dental pulp biomechanical behavior, and none assess NVB. Herein is the first to provide a clear picture of biomechanical stress distribution in the pulp and NVB during orthodontic movements performed in intact periodontium and under large forces (2–4 N). This aspect is clinically important in both conceiving the treatment plan as well as in assessing if the application of loads could or would induce ischemic and resorptive risks. The issue of previously traumatized/injured teeth, as well as the dental pulp in previous dental treatments involving the pulp, was never approached in numerical studies; thus, our study is the first to perform such correlations. The report that larger orthodontic forces could in fact induce resorptive and ischemic risks in these above-mentioned situations is clinically of extreme importance. From the biomechanical point of view, our study is the first to provide a correct and workable numerical analysis to investigate the pulp and NVB tissues, addressing all mandatory conditions that ensure the method’s accuracy in the engineering field.

## Figures and Tables

**Table 1:** Physical properties of materials.

Materials	Young’s Modulus, E (GPa)	Poisson Ratio, *v*	Refs.
Enamel	80	0.33	[4,5,37,38,39,42,43,44]
Dentin/Cementum	18.6	0.31	[4,5,37,38,39,42,43,44]
Pulp and NVB	0.0021	0.45	[4,5,37,38,39,42,43,44]
PDL	0.0667	0.49	[4,5,37,38,39,42,43,44]
Cortical bone	14.5	0.323	[4,5,37,38,39,42,43,44]
Trabecular bone	1.37	0.3	[4,5,37,38,39,42,43,44]
Stainless-streel bracket (Cr-Co)	218	0.33	[4,5,37,38,39,42,43,44]

**Table 2:** Shear stress average values (KPa) in intact periodontium for 2 and 4 N for the nine models.

Resorption (mm)		Rotation	Translation	Tipping	Extrusion	Intrusion
2 N	NVB	0.57	0.37	0.49	0.57	0.57
	% NVB	1.00	1.00	1.00	1.00	1.00
	pulp	0.05	0.03	0.04	0.05	0.05
	% pulp	1.00	1.00	1.00	1.00	1.00
4 N	NVB	1.15	0.74	0.98	1.14	1.14
	% NVB	1.00	1.00	1.00	1.00	1.00
	pulp	0.10	0.07	0.08	0.10	0.10
	% pulp	1.00	1.00	1.00	1.00	1.00

NVB—neurovascular bundle, % NVB—No. of times of stress increase, pulp—apical third, % pulp—No. of times of stress increase.

## Data Availability

The original contributions presented in the study are included in the article, further inquiries can be directed to the corresponding authors.

## References

[1] Cărămizaru M., Pleşea I.E., Dragomir L.P., Popescu M.R., Uscatu C.D., Şerbănescu M.S., Alexandru D.O., Comănescu T.M. (2018). Quantitative assessment of morphological changes of dental pulp components of teeth affected by occlusal trauma. Rom. J. Morphol. Embryol. =Rev. Roum. Morphol. Embryol..

[2] Rusu Olaru A., Popescu M.R., Pleşea I.E., Şerbănescu M.S., Pleşea R.M., Cojocaru M.O., Coculescu E.C. (2024). Abrasion and dental pulp morphological changes in occlusal dysfunction. Rom. J. Morphol. Embryol. = Rev. Roum. Morphol. Embryol..

[3] França C.M., Riggers R., Muschler J.L., Widbiller M., Lococo P.M., Diogenes A., Bertassoni L.E. (2019). 3D-Imaging of Whole Neuronal and Vascular Networks of the Human Dental Pulp via CLARITY and Light Sheet Microscopy. Sci. Rep..

[4] Moga R.A., Buru S.M., Olteanu C.D. (2022). Assessment of the Best FEA Failure Criteria (Part II): Investigation of the Biomechanical Behavior of Dental Pulp and Apical-Neuro-Vascular Bundle in Intact and Reduced Periodontium. Int. J. Environ. Res. Public Health.

[5] Moga R.A., Buru S.M., Chiorean C.G. (2022). Overall stress in periodontal ligament under orthodontic movement during a periodontal breakdown. Am. J. Orthod. Dentofac. Orthop. Off. Publ. Am. Assoc. Orthod. Const. Soc. American Board Orthod..

[6] Cox C.F., Hafez A.A. (2001). Biocomposition and reaction of pulp tissues to restorative treatments. Dent. Clin. N. Am..

[7] Wu J., Liu Y., Wang D., Zhang J., Dong X., Jiang X., Xu X. (2019). Investigation of effective intrusion and extrusion force for maxillary canine using finite element analysis. Comput. Methods Biomech. Biomed. Eng..

[8] Wu J., Liu Y., Li B., Wang D., Dong X., Sun Q., Chen G. (2021). Numerical simulation of optimal range of rotational moment for the mandibular lateral incisor, canine and first premolar based on biomechanical responses of periodontal ligaments: A case study. Clin. Oral Investig..

[9] Hohmann A., Wolfram U., Geiger M., Boryor A., Kober C., Sander C., Sander F.G. (2009). Correspondences of hydrostatic pressure in periodontal ligament with regions of root resorption: A clinical and a finite element study of the same human teeth. Comput. Methods Programs Biomed..

[10] Hohmann A., Wolfram U., Geiger M., Boryor A., Sander C., Faltin R., Faltin K., Sander F.G. (2007). Periodontal ligament hydrostatic pressure with areas of root resorption after application of a continuous torque moment. Angle Orthod..

[11] Weissheimer T., Silva E., Pinto K.P., Só G.B., Rosa R.A., Só M.V.R. (2021). Do orthodontic tooth movements induce pulp necrosis? A systematic review. Int. Endod. J..

[12] Yamaguchi M., Fukasawa S. (2021). Is Inflammation a Friend or Foe for Orthodontic Treatment?: Inflammation in Orthodontically Induced Inflammatory Root Resorption and Accelerating Tooth Movement. Int. J. Mol. Sci..

[13] Ricucci D., Siqueira J.F., Rôças I.N. (2021). Pulp Response to Periodontal Disease: Novel Observations Help Clarify the Processes of Tissue Breakdown and Infection. J. Endod..

[14] Minch L.E., Sarul M., Nowak R., Kawala B., Antoszewska-Smith J. (2017). Orthodontic intrusion of periodontally-compromised maxillary incisors: 3-dimensional finite element method analysis. Adv. Clin. Exp. Med. Off. Organ Wroc. Med. Univ..

[15] Bauss O., Rohling J., Rahman A., Kiliaridis S. (2008). The effect of pulp obliteration on pulpal vitality of orthodontically intruded traumatized teeth. J. Endod..

[16] Bauss O., Röhling J., Sadat-Khonsari R., Kiliaridis S. (2008). Influence of orthodontic intrusion on pulpal vitality of previously traumatized maxillary permanent incisors. Am. J. Orthod. Dentofac. Orthop. Off. Publ. Am. Assoc. Orthod. Const. Soc. Am. Board Orthod..

[17] Bauss O., Schäfer W., Sadat-Khonsari R., Knösel M. (2010). Influence of orthodontic extrusion on pulpal vitality of traumatized maxillary incisors. J. Endod..

[18] Bauss O., Rohling J., Meyer K., Kiliaridis S. (2009). Pulp vitality in teeth suffering trauma during orthodontic therapy. Angle Orthod..

[19] Cardenas-Duque L.M., Yoshida M., Goto G. (2002). Pulpal response to different pulp capping methods after pulp exposure by air abrasion. J. Clin. Pediatr. Dent..

[20] Murray P.E., Hafez A.A., Windsor L.J., Smith A.J., Cox C.F. (2002). Comparison of pulp responses following restoration of exposed and non-exposed cavities. J. Dent..

[21] Mjör I.A. (2002). Pulp-dentin biology in restorative dentistry. Part 7: The exposed pulp. Quintessence Int..

[22] Murray P.E., Hafez A.A., Smith A.J., Cox C.F. (2003). Identification of hierarchical factors to guide clinical decision making for successful long-term pulp capping. Quintessence Int..

[23] Kitasako Y., Murray P.E., Tagami J., Smith A.J. (2002). Histomorphometric analysis of dentinal bridge formation and pulpal inflammation. Quintessence Int..

[24] Medina V.O., Shinkai K., Shirono M., Tanaka N., Katoh Y. (2002). Histopathologic study on pulp response to single-bottle and self-etching adhesive systems. Oper. Dent..

[25] Suzuki M., Katsumi A., Watanabe R., Shirono M., Katoh Y. (2005). Effects of an experimentally developed adhesive resin system and CO_2_ laser irradiation on direct pulp capping. Oper. Dent..

[26] Kitasako Y., Ikeda M., Tagami J. (2008). Pulpal responses to bacterial contamination following dentin bridging beneath hard-setting calcium hydroxide and self-etching adhesive resin system. Dent. Traumatol. Off. Publ. Int. Assoc. Dent. Traumatol..

[27] Schuurs A.H., Gruythuysen R.J., Wesselink P.R. (2000). Pulp capping with adhesive resin-based composite vs. calcium hydroxide: A review. Endod. Dent. Traumatol..

[28] Farughi A., Rouhani A., Shahmohammadi R., Jafarzadeh H. (2021). Clinical comparison of sensitivity and specificity between sensibility and vitality tests in determining the pulp vitality of mandibular premolars. Aust. Endod. J. J. Aust. Soc. Endodontol. Inc..

[29] Patro S., Meto A., Mohanty A., Chopra V., Miglani S., Das A., Luke A.M., Hadi D.A., Meto A., Fiorillo L. (2022). Diagnostic Accuracy of Pulp Vitality Tests and Pulp Sensibility Tests for Assessing Pulpal Health in Permanent Teeth: A Systematic Review and Meta-Analysis. Int. J. Environ. Res. Public Health.

[30] Javed F., Al-Kheraif A.A., Romanos E.B., Romanos G.E. (2015). Influence of orthodontic forces on human dental pulp: A systematic review. Arch. Oral Biol..

[31] Strobl H., Haas M., Norer B., Gerhard S., Emshoff R. (2004). Evaluation of pulpal blood flow after tooth splinting of luxated permanent maxillary incisors. Dent. Traumatol. Off. Publ. Int. Assoc. Dent. Traumatol..

[32] Emshoff R., Emshoff I., Moschen I., Strobl H. (2004). Diagnostic characteristics of pulpal blood flow levels associated with adverse outcomes of luxated permanent maxillary incisors. Dent. Traumatol. Off. Publ. Int. Assoc. Dent. Traumatol..

[33] Chen E., Abbott P.V. (2009). Dental pulp testing: A review. Int. J. Dent..

[34] Balevi B. (2019). Cold pulp testing is the simplest and most accurate of all dental pulp sensibility tests. Evid.-Based Dent..

[35] Mainkar A., Kim S.G. (2018). Diagnostic Accuracy of 5 Dental Pulp Tests: A Systematic Review and Meta-analysis. J. Endod..

[36] Proffit W.R., Fields H., Sarver D.M., Ackerman J.L. (2012). Contemporary orthodontics.

[37] Moga R.A., Delean A.G., Buru S.M., Botez M.D., Olteanu C.D. (2023). Orthodontic Internal Resorption Assessment in Periodontal Breakdown-A Finite Elements Analysis (Part II). Healthcare.

[38] Moga R.A., Olteanu C.D., Botez M.D., Buru S.M. (2023). Assessment of the Orthodontic External Resorption in Periodontal Breakdown-A Finite Elements Analysis (Part I). Healthcare.

[39] Moga R.A., Olteanu C.D., Botez M., Buru S.M. (2023). Assessment of the Maximum Amount of Orthodontic Force for Dental Pulp and Apical Neuro-Vascular Bundle in Intact and Reduced Periodontium on Bicuspids (Part II). Int. J. Environ. Res. Public Health.

[40] Vitali F.C., Cardoso I.V., Mello F.W., Flores-Mir C., Andrada A.C., Dutra-Horstmann K.L., Duque T.M. (2021). Effect of orthodontic force on dental pulp histomorphology and tissue factor expression. Angle Orthod..

[41] Vermiglio G., Centofanti A., Matarese G., Militi A., Matarese M., Arco A., Nicita F., Cutroneo G. (2020). Human Dental Pulp Tissue during Orthodontic Tooth Movement: An Immunofluorescence Study. J. Funct. Morphol. Kinesiol..

[42] Moga R.-A., Olteanu C.D., Delean A.G. (2024). Investigating the Ability of the Tooth and Surrounding Support Tissues to Absorb and Dissipate Orthodontic Loads during Periodontal Breakdown&mdash;Finite Elements Analysis. Appl. Sci..

[43] Moga R.A., Olteanu C.D., Buru S.M., Botez M.D., Delean A.G. (2023). Finite Elements Analysis of Biomechanical Behavior of the Bracket in a Gradual Horizontal Periodontal Breakdown&mdash;A Comparative Analysis of Multiple Failure Criteria. Appl. Sci..

[44] Moga R.A., Olteanu C.D., Buru S.M., Botez M.D., Delean A.G. (2023). Cortical and Trabecular Bone Stress Assessment during Periodontal Breakdown-A Comparative Finite Element Analysis of Multiple Failure Criteria. Medicina.

[45] Wu J.L., Liu Y.F., Peng W., Dong H.Y., Zhang J.X. (2018). A biomechanical case study on the optimal orthodontic force on the maxillary canine tooth based on finite element analysis. J. Zhejiang Univ. Sci. B.

[46] Perez-Gonzalez A., Iserte-Vilar J.L., Gonzalez-Lluch C. (2011). Interpreting finite element results for brittle materials in endodontic restorations. Biomed. Eng. Online.

[47] Toms S.R., Eberhardt A.W. (2003). A nonlinear finite element analysis of the periodontal ligament under orthodontic tooth loading. Am. J. Orthod. Dentofac. Orthop. Off. Publ. Am. Assoc. Orthod. Const. Soc. Am. Board Orthod..

[48] Hemanth M., Deoli S., Raghuveer H.P., Rani M.S., Hegde C., Vedavathi B. (2015). Stress Induced in the Periodontal Ligament under Orthodontic Loading (Part I): A Finite Element Method Study Using Linear Analysis. J. Int. Oral Health JIOH.

[49] Hemanth M., Deoli S., Raghuveer H.P., Rani M.S., Hegde C., Vedavathi B. (2015). Stress Induced in Periodontal Ligament under Orthodontic Loading (Part II): A Comparison of Linear Versus Non-Linear Fem Study. J. Int. Oral Health JIOH.

[50] Geramy A. (2002). Initial stress produced in the periodontal membrane by orthodontic loads in the presence of varying loss of alveolar bone: A three-dimensional finite element analysis. Eur. J. Orthod..

[51] Geramy A., Faghihi S. (2004). Secondary trauma from occlusion: Three-dimensional analysis using the finite element method. Quintessence Int..

[52] Shaw A.M., Sameshima G.T., Vu H.V. (2004). Mechanical stress generated by orthodontic forces on apical root cementum: A finite element model. Orthod. Craniofacial Res..

[53] Gupta M., Madhok K., Kulshrestha R., Chain S., Kaur H., Yadav A. (2020). Determination of stress distribution on periodontal ligament and alveolar bone by various tooth movements—A 3D FEM study. J. Oral Biol. Craniofacial Res..

[54] Merdji A., Mootanah R., Bachir Bouiadjra B.A., Benaissa A., Aminallah L., Ould Chikh el B., Mukdadi S. (2013). Stress analysis in single molar tooth. Mater. Sci. Eng. C Mater. Biol. Appl..

[55] Roscoe M.G., Cattaneo P.M., Dalstra M., Ugarte O.M., Meira J.B.C. (2021). Orthodontically induced root resorption: A critical analysis of finite element studies’ input and output. Am. J. Orthod. Dentofac. Orthop. Off. Publ. Am. Assoc. Orthod. Const. Soc. Am. Board Orthod..

[56] Prados-Privado M., Martínez-Martínez C., Gehrke S.A., Prados-Frutos J.C. (2020). Influence of Bone Definition and Finite Element Parameters in Bone and Dental Implants Stress: A Literature Review. Biology.

[57] Yamanishi Y., Yamaguchi S., Imazato S., Nakano T., Yatani H. (2014). Effects of the implant design on peri-implant bone stress and abutment micromovement: Three-dimensional finite element analysis of original computer-aided design models. J. Periodontol..

[58] Pérez-Pevida E., Brizuela-Velasco A., Chávarri-Prado D., Jiménez-Garrudo A., Sánchez-Lasheras F., Solaberrieta-Méndez E., Diéguez-Pereira M., Fernández-González F.J., Dehesa-Ibarra B., Monticelli F. (2016). Biomechanical Consequences of the Elastic Properties of Dental Implant Alloys on the Supporting Bone: Finite Element Analysis. BioMed Res. Int..

[59] Aunmeungtong W., Khongkhunthian P., Rungsiyakull P. (2016). Stress and strain distribution in three different mini dental implant designs using in implant retained overdenture: A finite element analysis study. ORAL Implantol..

[60] Merdji A., Bachir Bouiadjra B., Achour T., Serier B., Ould Chikh B., Feng Z.O. (2010). Stress analysis in dental prosthesis. Comput. Mater. Sci..

[61] Field C., Ichim I., Swain M.V., Chan E., Darendeliler M.A., Li W., Li Q. (2009). Mechanical responses to orthodontic loading: A 3-dimensional finite element multi-tooth model. Am. J. Orthod. Dentofac. Orthop. Off. Publ. Am. Assoc. Orthod. Const. Soc. Am. Board Orthod..

[62] Shetty B., Fazal I., Khan S.F. (2022). FEA analysis of Normofunctional forces on periodontal elements in different angulations. Bioinformation.

